# Trends and Determinants of Child Growth Indicators in Malawi and Implications for the Sustainable Development Goals

**DOI:** 10.3934/publichealth.2017.6.590

**Published:** 2017-11-30

**Authors:** Henry V Doctor, Sangwani Nkhana-Salimu

**Affiliations:** 1 Regional Office for the Eastern Mediterranean, World Health Organization, Cairo, Egypt;; 2 College of Medicine, School of Public Health, University of Malawi, Blantyre 30096, Malawi

**Keywords:** malnutrition, overweight, stunting, underweight, wasting, sub-Saharan Africa

## Abstract

Sustainable development goals (SGD) 2 links malnutrition, morbidity and child mortality to stunting, wasting and overweight among children under-5 years of age. Sub-Saharan Africa still registers high nutritionally insecure people. In particular, Malawi has made modest progress in improving nutritional outcomes; and still experiences a number of structural challenges leading to negative nutritional outcomes. We describe trends of under nutrition and how the effect of selected determinants of child nutrition affect Malawian children under-5 from 1992 to 2015–16; and examine the changing patterns of the effect of selected socio-demographic characteristics on stunting and underweight using data from demographic and health surveys (DHS). The analysis included 31,630 children under-5 years from 1992, 2000, 2004, 2010, and 2015–16 DHS. Our outcome measures are stunting (height/length-for-age) and underweight (weight-for-age) less than -2 SD (Z-score). We perform logistic regression to assess the relationship between selected socio-demographic characteristics with the stunting and underweight variables. Underweight decreased by 14.0% from 24.7% (1992) to 10.7% (2015–16). Stunting decreased by 23.0% from 55.6% (1992) to 32.6% (2015–16). Underweight was more prevalent among children from central and southern regions; among male children; and children above 6 months of age or more. Later surveys were associated with reduced likelihood of underweight than the earliest surveys. Similar trends were observed between socioeconomic factors and stunting. The observed underweight and stunting prevalence is 2.2% and 1.9% lower than expected, respectively. Despite modest declines in underweight and stunting among young children in Malawi, underweight and stunting remain significant public health challenges particularly in southern and central Malawi which constitute about 85% of the total population. Interventions to address the critical malnutrition challenges in Malawi are inevitable within the context of SDG 2 on health.

## 1. Introduction

The role of nutrition as a key driver of economic development has been well recognized. Nutrition serves as a foundation for healthy individuals and societies. By implication, malnutrition impedes human performance, health complications and human survival. Children who are well nourished are able to maximize their potential in expected growth and contribute to socio-economic development over the course of their life. The link between malnutrition, morbidity and child mortality has been highlighted in earlier studies [1] and is the focus of sustainable development goal (SGD) 2, target 2.2 calling for strategies to end all forms of malnutrition focused on stunting, wasting and overweight among children under-5 years of age [2].

Poor nutrition in the first 1,000 days of life impedes cognitive development, schooling and work performance [3] thereby lowering adult incomes and ultimately compromising national development [1]. Malnutrition is also one of the major challenges for child health in sub-Saharan Africa; and globally, more than one-third of postneonatal child deaths are attributable to undernutrition [4]. In 2015, sub-Saharan Africa was one of the two regions that accounted for almost 90% of underweight children; half were in Southern Asia and one third in sub-Saharan Africa. Despite a number of interventions to address malnutrition in sub-Saharan Africa since the 1990s, eradicating poverty and hunger remains a challenge and is at the core of the post-2015 development agenda. Many of the people suffering the most live in settings with protracted emergencies and poor access to life enhancing resources [5].

Malawi is no exception with regard to nutrition challenges, despite national and international efforts to address the problem. The country has experienced a persistent rural-urban malnutrition gap, with the prevalence of malnutrition being higher in the rural areas than in the urban areas [6]. Although Malawi was able to make economic and structural reforms and sustain its economic growth rates since 2005, poverty is still widespread and the economy remains undiversified and vulnerable to external shocks. Key challenges to growth in real gross domestic product have largely been related to severe drought and flooding in the southern part of the country; and affected agricultural production. The country registered an average 30.2% year-on-year drop in maize (key crop for food security purposes) since 2014. The Malawi Vulnerability Assessment Committee estimated that in 2016 about 7 million people would require food assistance [7].

A number of studies conducted previously have identified the magnitude and determinants of undernutrition in Malawi [6],[8],[9]. What lacks, however, are studies that examine trends of undernutrition and how the effect of selected determinants of child nutrition status changes over time. We bridge this gap by describing trends in underweight and stunting among Malawian children for slightly over two decades (1992–2015); and examine the changing patterns of the effect of selected socio-demographic characteristics on child nutritional status (stunting and underweight) using data from Demographic and Health Surveys (DHS). The motivation to select these two measures is two-fold: ➀ stunting is a better measure than underweight of the cumulative effects of undernutrition and infection during the critical 1,000-d period from pregnancy to the child's second or third birthday [5]; and ➁ stunting is also more common than underweight, affecting more than one in four children under-5 or 161 million children worldwide in 2013 [5]. Being underweight also puts children at greater risk of dying from common infections, increases the frequency and severity of such infections and contributes to delayed recovery. Therefore, understanding trends and determinants in stunting and underweight can inform strategies to address public health challenges of malnutrition and ensure that no child is left behind by 2030, the deadline to meet the SDGs.

## 2. Materials and Methods

### 2.1. Data

We analysed publicly de-identified data from five DHSs (http://www.dhsprogram.com/) conducted in 1992, 2000, 2004, 2010, and 2015–16. The DHS are nationally representative population-based surveys with a historical focus on fertility and reproductive health but also covers a variety of child health outcomes and household characteristics. The DHS program is largely funded by the U.S. Agency for International Development. The DHS utilizes a multistage stratified cluster sampling methodology in which samples of households within clusters (enumeration areas) are selected. Households then are systematically selected within each cluster and household residents are eligible to participate in the survey. Urban areas are oversampled and our analysis was based on weighted data to account for the different sample proportions. A total of 5 811 households were selected for inclusion in the 1992 DHS, 15 421 in 2000, 15 041 in 2004, 27 307 in 2010 and 27 516 in 2015–16, with an average household response rate of 98.5% for all the surveys. The analysis included children under-5 years of age whose weight and height/length measurements were taken. Therefore, this analysis included 3 293, 9 651, 8 752, 4 791 and 5 143 Malawian children under-5 years in 1992, 2000, 2004, 2010 and 2015, respectively.

### 2.2. Undernutrition measurements and socio-demographic characteristics

Our measures were stunting and underweight, defined as height/length-for-age and weight-for-age less than -2 SD (Z-score) defined by the WHO growth reference standards for children of the same age and sex [10]. Simply put, stunting is defined as inadequate height for age whereas underweight is body weight considered too low to be healthy. We pool all the DHS data (*n*=31,630 children) and use the two dependent variables: stunting (yes, no) and underweight (yes, no) in a logistic regression to assess the relationship between selected socio-demographic characteristics with the stunting and underweight variables. Table 1 provides a description of the selected measures in the study and how the characteristics were measured. Analyses were conducted *via* survey procedures including weights to account for the multistage survey design used in the DHS. The distribution of children by stunting and underweight were calculated using chi-square contingency tables and applying weighted frequencies. Statistical significance was assessed at *P* < 0.05.

**Table 1.** Description of characteristics and their measures used in the analysis of stunting and underweight, Malawi Demographic and Health Survey, 1992, 2000, 2004, 2010, 2015–16.

**Measure**	**Categories used**	**Description**
**Household characteristics**		
Residence	Urban, rural	Based on the sample allocation of respondents for each sampled area
Region of residence	Northern, central, southern	Based on the sample allocation of respondents for each sampled area
Wealth index	Poorest, poor, middle, richer, and richest	The household asset index for is for the household to which the respondent belongs
Source of drinking water	Non-improved, improved	Improved sources for the household included piped water, tap water, bottled water and protected wells in the compound. Unprotected wells, springs, rivers, ponds, lakes and dams were grouped as non-improved water sources.
Toilet facilities	No toilet facility, Non-improved, Improved	Improved household toilet facilities included flush toilets and ventilated improved pit latrines (VIP) latrines, while traditional pit latrines were categorized as non-improved toilet facilities
**Maternal characteristics**		
Highest educational level	No education, Primary, Secondary and above	Respondent was asked if she had ever attended school, and if so, what were the highest level and class that she completed
Mother's age	<20 years, 20–30 years, >30 years	Based on respondent's reports of age in completed years
Number of under-5 children living with mother	1, 2–3, 4–5, 6 and above	Based on reports of number of children under-5 living with the mother
**Child-related characteristics**		
Sex	Male, female	Respondents reports of the sex of the child
Age (months)	≤ 6, 7–12, 13–23, 24–35, 36–59	Respondents reports of the age of child at the time of the survey
Birth order	1, 2–3, ≥ 4	Birth order of the child for children born 5 years prior to the survey
Size at birth	Small, average, large	Respondent's reports of weight of child at birth, with a reference weight of 2.5 kg as average weight
Had diarrhoea	Yes, no	Respondent report whether child had diarrhoea in the last two weeks before the survey
Had fever	Yes, no	Respondent report whether child had fever in the last two weeks before the survey
Had cough	Yes, no	Respondent report whether child had cough in the last two weeks before the survey

## 3. Results

**Figure 1.**
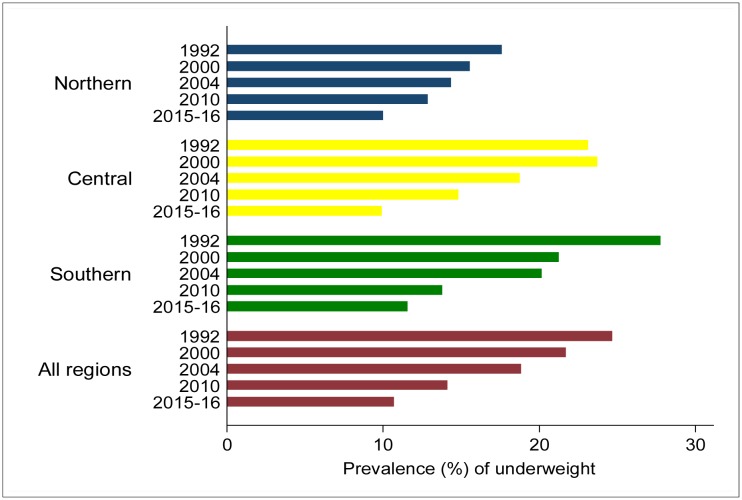
Prevalence of underweight among children under-5 years by year of survey and region of residence, Malawi demographic and health surveys conducted from 1990 to 2015–16.

**Figure 2.**
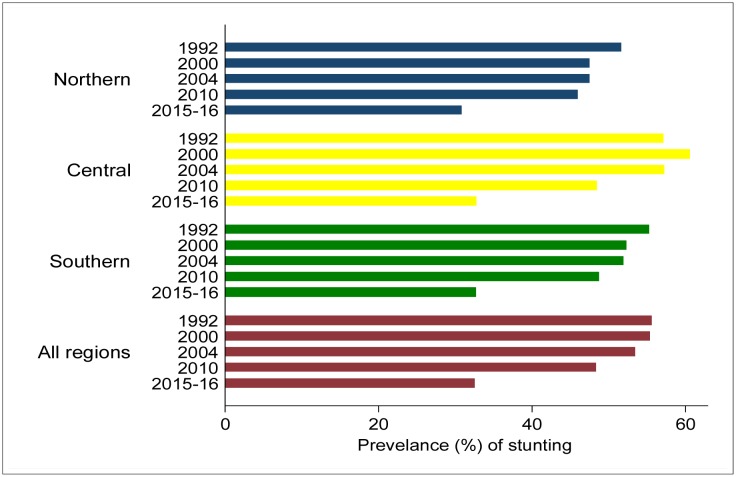
Prevalence of stunting among children under-5 years by year of survey and region of residence, Malawi demographic and health surveys conducted from 1990 to 2015–16.

The study included children aged 0–59 months with a mean age of 27.1 months in 1992; 26.8 months in 2000; 27.2 months in 2004; 29.0 months in 2010; and 29.3 months in 2015–16. The overall mean age for all the children (*n*=31,630) was 27.7 months. The mean height and weight of the children in all the surveys was 80.2 cm and 10.8 kg, respectively. There were no significant age differences by child's gender. The mean weight-for-age Z-score increased from -1.1 in 1992 to -0.8 in 2015–16. The prevalence of underweight decreased by 14.0 percentage points from 24.7% in 1992 to 10.7% in 2015–16 (Figure 1). The mean height-for-age Z-score increased from -2.1 in 1992 to -1.4 in 2015–16. The prevalence of stunting decreased by 23.0 percentage points from 55.6% in 1992 to 32.6% in 2015–16 (Figure 2).

At least 85% of children in each of the survey period lived in urban areas. Except for 2010, nearly half of children were sampled from Southern region followed by Central region and Northern region (Table 2). About 43% of all the households were below the middle wealth group and did not vary a great deal during the period. Nearly half (47.7%) of households used improved sources of water in 1992 and declined to 10.7% in 2015–16, with some fluctuations during the period. Across all surveys, use of improved sources of water among households was 28.5%. The proportion of households with no toilet facilities declined from 25.8% in 1992 to 5.7% in 2015–16, with 15.1% of households across the surveys without toilet facilities. Children of mothers with at least secondary schooling increased from 2.9% in 1992 to 20.7% in 2015–16; with 10.8% of mothers reporting at least secondary schooling across the surveys. The proportion of young mothers (<20 years) remained almost stable, at 7.6% in 1992 and 6.9% in 2015–16. The proportion of mothers living with at least 6 children under-5 years declined from 20.3% in 1992 to 11.6% in 2015–16. The sex ratio of children (male/female) declined from 1.01 in 1992 to 0.94 in 2015–16, with an overall sex ratio of 0.98 across the years. Children aged 36–59 months constituted the highest proportion across the survey years (36.1%); and the same trend for children with birth order 2–3, at 53.5% in the pooled data.

**Table 2.** Distribution of household, maternal and child-related characteristics: Malawi demographic and health survey, 1992, 2000, 2004, 2010, 2015–16 *n* (%).

Characteristics	1992 (*n*=3,293)	2000 (*n*=9,651)	2004 (*n*=8,752)	2010 (*n*=4,791)	2015–16 (*n*=5,143)	All years (*n*=31,630)
**Household characteristics**						
Residence						
Urban	351 (10.7)	1,242 (13.0)	1,108 (12.7)	718 (15.0)	661 (12.8)	4,086 (12.9)
Rural	2,942 (89.3)	8,401 (87.1)	7,644 (87.3)	4,073 (85.0)	4,482 (87.2)	27,544 (87.1)
Region						
Northern	383 (11.6)	1,051 (10.9)	1,158 (13.2)	534 (11.2)	546 (10.6)	3,668 (11.6)
Central	1,358 (41.2)	4,205 (43.6)	3,492 (39.9)	2,188 (45.7)	2,194 (42.7)	13,443 (42.5)
Southern	1,552 (47.1)	4,395 (45.5)	4,102 (46.9)	2,069 (43.2)	2,402 (46.7)	14,519 (45.9)
Wealth index						
Poorest	685 (20.8)	2,253 (23.4)	1,709 (19.5)	859 (17.9)	1,238 (24.1)	6,754 (21.4)
Poor	624 (18.9)	2,026 (21.0)	1,915 (21.9)	1,073 (22.4)	1,208 (23.5)	6,842 (21.6)
Middle	731 (22.2)	1,951 (20.2)	1,983 (22.7)	1,076 (22.5)	992 (19.3)	6,728 (21.3)
Richer	684 (20.8)	1,765 (18.3)	1,769 (20.2)	873 (18.2)	903 (17.6)	5,991 (18.9)
Richest	568 (17.3)	1,656 (17.2)	1,376 (15.7)	910 (19.0)	802 (15.6)	5,315 (16.8)
**Source of drinking water**						
Non-improved	1,717 (52.3)	7,565 (78.4)	5,162 (59.0)	3,727 (77.8)	3,732 (89.3)	21,931 (71.6)
Improved	1,568 (47.7)	2,082 (21.6)	3,590 (41.0)	1,064 (22.2)	445 (10.7)	8,721 (28.5)
Toilet facilities						
No toilet facility	847 (25.8)	1,756 (18.2)	1,389 (15.9)	473 (9.9)	292 (5.7)	4,772 (15.1)
Non-improved	2,355 (71.6)	7,645 (79.3)	7,119 (81.3)	3,831 (80.0)	773 (15.1)	21,713 (68.7)
Improved	86 (2.6)	240 (2.5)	244 (2.8)	487 (10.2)	4,066 (79.3)	5,117 (16.2)
**Maternal characteristics**						
Highest education level						
No education	1,708 (51.9)	3,099 (32.1)	2,224 (25.4)	824 (17.2)	696 (13.5)	8,585 (27.1)
Primary	1,489 (45.2)	5,908 (61.2)	5,609 (64.1)	3,253 (67.9)	3,383 (65.8)	19,622 (62.0)
Secondary+	96.8 (2.9)	644 (6.7)	919 (10.5)	714 (14.9)	1,064 (20.7)	3,423 (10.8)
Mothers age (yr)						
<20	250 (7.6)	661 (6.9)	567 (6.5)	271 (5.7)	355 (6.9)	2,105 (6.7)
20–30	1,563 (47.5)	5,549 (57.5)	5,178 (59.2)	2,753 (57.5)	2,825 (54.9)	17,587 (56.5)
>30	1,481 (45.0)	3,441(35.7)	3,007 (34.4)	1,768 (36.9)	1,964 (38.2)	11,668 (36.9)
Number of under-5 children living with mother						
1	484 (14.9)	1,825 (19.1)	1,453 (16.7)	690 (14.5)	1,030 (20.1)	5,487 (17.5)
2–3	1,190 (36.7)	4,038 (42.2)	3,934 (45.3)	2,124 (44.6)	2,238 (43.7)	13,513 (43.1)
4–5	907 (28.0)	2,302 (24.1)	2,127 (24.5)	1,202 (25.2)	1,261 (24.6)	7,802 (24.9)
6+	660 (20.3)	1,397 (14.6)	1,174 (13.5)	748 (15.7)	592 (11.6)	4,577 (14.6)
**Child-related characteristics**						
Sex						
Male	1,652 (50.2)	4,752 (49.2)	4,392 (50.2)	2,346 (49.0)	2,494 (48.5)	15,635 (49.4)
Female	1,641 (49.8)	4,899 (50.8)	4,360 (49.8)	2,445 (51.0)	2,649 (51.5)	15,995 (50.6)
Age (mo)						
≤6	485 (14.7)	1,361 (14.1)	1,125 (12.9)	513 (10.7)	573 (11.2)	4,063 (12.9)
7–12	428 (13.0)	1,180 (12.2)	1,098 (12.5)	494 (10.3)	560 (10.9)	3,760 (11.9)
13–23	632 (19.2)	1,892 (19.6)	1,887 (21.6)	1,010 (21.1)	913 (17.8)	6,329 (20.0)
24–35	593 (18.0)	1,936 (20.1)	1,543 (17.6)	958 (20.0)	1,028 (20.0)	6,062 (19.2)
36–59	1,155 (35.1)	3,282 (34.0)	3,098 (35.4)	1,816 (37.9)	2,068 (40.2)	11,419 (36.1)
Birth order						
1	558 (17.1)	1,493 (15.5)	1,275 (15.0)	644 (13.7)	835 (16.4)	4,808 (15.4)
2–3	2,070 (63.6)	5,705 (59.4)	4,308 (50.5)	2,030 (43.3)	2,530 (49.6)	16,671 (53.5)
≥4	629 (19.3)	2,411 (25.1)	2,945 (34.5)	2,015 (43.0)	1,735 (34.0)	9,704 (31.1)
Size at birth						
Small	557 (17.1)	1,493 (15.5)	1,275 (15.0)	644 (13.7)	835 (16.4)	4,808 (15.4)
Average	2,070 (63.6)	5,705 (59.4)	4,308 (50.5)	2,030 (43.3)	2,530 (49.6)	16,671 (53.5)
Large	629 (19.3)	2,411 (25.1)	2,945 (34.5)	2,015 (43.0)	1,735 (34.0)	9,704 (31.1)
Diarrhoea						
No	2,540 (77.2)	7,887 (81.8)	6,732 (76.9)	4,045 (84.6)	4,034 (78.4)	25,244 (79.9)
Yes	749 (22.8)	1,761 (18.3)	2,017 (23.1)	739 (15.5)	1,109 (21.6)	6,369 (20.2)
Fever						
No	1,941 (59.0)	5,525 (57.3)	5,400 (61.7)	3,186 (66.6)	3,552 (69.1)	19,590 (62.0)
Yes	1,348 (41.0)	4,122 (42.7)	3,349 (38.3)	1,598 (33.4)	1,591 (30.9)	12,022 (38.0)
Cough						
No	1,891 (57.5)	5,034 (52.2)	5,350 (61.2)	3,453 (72.6)	3,830 (74.5)	19,532 (61.8)
Yes	1,396 (42.5)	4,614 (47.8)	3,398 (38.8)	1,306 (27.4)	1,313 (25.5)	12,053 (38.2)

Note: Some percentages may not add up to 100 due to rounding of figures.

**Table 3.** Percentage of underweight and stunting among children under-5 years by household, maternal and child-relate characteristics, Malawi demographic and health survey, 1992, 2000, 2004, 2010, 2015–16.

Characteristics	Underweight (weight-for-age < -2 SD)					Stunting (height-for-age < -2 SD)				
	1992	2000	2004	2010	2015–16	1992	2000	2004	2010	2015–16
Total	24.7	21.7	18.8	14.1	10.7	55.6	55.4	53.5	48.4	32.6
95%CI	22.6, 26.7	20.5, 22.9	17,7, 20.0	12.8, 15.5	9.7, 11.8	53.5, 57.7	53.8, 57.0	51.9, 55.0	46.4, 50.3	30.9, 34.3
Standard Error	0.0104	0.0063	0.0057	0.0071	0.0054	0.0107	0.0081	0.0077	0.0100	0.0088
Household characteristics										
Residence										
Urban	15.4	13.3	12.8	11.7	6.7	4.5	5.4	5.6	6.3	2.9
Rural	25.8	22.9	19.9	14.6	11.3	51.1	50.0	47.9	42.1	29.7
*P* value	0.000	0.000	0.001	0.241	0.007	0.000	0.000	0.000	0.001	0.000
Region										
Northern	17.6	15.6	14.4	12.9	10.0	6.2	5.3	6.4	4.9	3.4
Central	23.2	23.7	18.8	14.8	9.9	23.5	26.2	22.5	22.4	14.1
Southern	27.8	21.3	20.2	13.8	11.6	25.9	23.9	24.5	21.1	15.2
*P* value	0.001	0.000	0.002	0.613	0.281	0.203	0.000	0.000	0.700	0.760
Wealth index										
Poorest	30.9	27.6	25.8	17.5	14.2	12.9	14.8	11.5	10.1	9.6
Poor	29.0	24.9	20.7	15.7	11.8	11.1	21.0	12.4	11.7	8.6
Middle	25.4	22.1	19.8	13.3	10.9	12.3	20.2	13.1	10.7	6.2
Richer	20.5	19.2	16.8	15.5	9.3	11.6	18.2	10.2	8.8	5.0
Richest	16.7	11.9	9.0	8.9	5.1	7.6	17.3	6.3	6.9	3.2
*P* value	0.000	0.000	0.000	0.003	0.000	0.000	0.000	0.000	0.000	0.000
Source of drinking water										
Non-improved	26.6	23.5	19.0	15.1	10.6	30.1	45.4	31.7	38.4	30.2
Improved	22.4	15.3	18.6	10.9	5.8	25.4	10.1	21.7	10.0	2.1
*P* value	0.040	0.000	0.733	0.034	0.020	0.020	0.000	0.350	0.043	0.000
Toilet facilities										
No toilet facility	29.9	27.4	24.2	19.9	19.9	15.3	11.2	9.6	5.3	1.9
Non-improved	23.5	20.8	18.2	13.9	13.9	39.3	43.4	42.7	39.0	5.2
Improved	4.3	10.8	7.7	10.2	10.2	9.3	0.8	1.2	4.2	25.5
*P* value	0.000	0.000	0.000	0.003	0.003	0.000	0.000	0.000	0.003	0.400
Maternal characteristics										
Highest education level										
No education	27.9	24.6	22.6	16.8	13.6	30.0	19.3	14.4	9.1	5.3
Primary	22.0	21.5	18.6	14.7	11.2	24.9	33.8	34.8	33.4	22.3
Secondary+	9.3	9.7	11.2	8.6	7.4	0.8	2.3	4.3	5.9	5.0
*P* value	0.000	0.000	0.000	0.002	0.002	0.000	0.000	0.000	0.000	0.000
Mothers age (yr)										
<20	28.6	26.4	19.5	19.3	8.6	4.0	3.3	2.9	2.1	1.9
20–30	23.2	20.9	17.7	13.4	10.5	25.9	57.4	31.8	28.3	18.5
>30	25.6	22.2	20.6	14.5	11.5	25.7	20.6	18.8	18.0	12.3
*P* value	0.159	0.025	0.037	0.134	0.362	0.164	0.012	0.018	0.030	0.131
Number of under-5 children living with mother										
1	24.9	23.4	18.4	16.9	9.9	7.3	10.1	8.2	6.8	5.8
2–3	23.3	21.1	17.5	12.9	9.6	20.4	23.5	24.6	21.5	14.4
4–5	27.7	21.3	20.6	14.3	12.1	17.0	13.5	13.2	12.4	8.5
≥6	22.5	21.8	20.7	14.5	13.6	10.8	8.4	7.6	7.5	3.9
*P* value	0.159	0.419	0.055	0.281	0.073	0.006	0.536	0.057	0.960	0.099
Child-related characteristics										
Sex										
Male	27.1	23.3	19.9	15.0	11.9	29.1	28.3	27.7	25.4	16.6
Female	22.2	20.1	17.8	13.3	9.6	26.5	27.1	25.8	23.0	16.0
*P* value	0.007	0.001	0.032	0.220	0.011	0.005	0.001	0.004	0.000	0.061
Age (mo)										
≤6	13.8	17.9	10.5	9.5	5.5	3.2	3.4	2.9	2.5	1.7
7–12	25.0	24.4	22.1	13.1	11.0	4.3	4.7	5.1	2.9	2.0
13–23	28.1	25.7	20.7	16.9	8.9	10.7	11.6	13.0	12.2	5.7
24–35	29.0	24.8	19.9	15.0	11.0	13.4	13.6	10.9	11.6	8.1
36–59	25.1	18.2	19.1	13.8	12.7	23.9	22.1	21.6	19.3	15.1
*P* value	0.000	0.000	0.000	0.039	0.002	0.000	0.000	0.000	0.000	0.000
Birth order										
1	24.0	226	18.0	16.2	10.5	8.9	12.2	11.4	9.4	8.0
2–3	23.4	20.6	17.8	12.9	9.1	15.7	19.2	19.7	17.9	12.2
≥4	25.6	22.2	20.2	14.3	12.6	31.0	24.0	22.3	21.1	12.4
*P* value	0.472	0.206	0.065	0.209	0.014	0.318	0.316	0.316	0.910	0.581
Size at birth										
Small	33.0	32.8	30.5	26.6	18.3	10.7	9.9	8.7	8.6	6.8
Average	23.9	21.6	18.0	14.0	10.5	35.5	33.0	27.0	21.2	15.6
Large	19.6	15.2	14.5	10.4	7.6	9.3	12.5	17.7	18.4	10.4
*P* value	0.000	0.000	0.000	0.000	0.000	0.000	0.000	0.000	0.000	0.000
Diarrhoea										
No	23.3	20.2	17.0	13.6	23.3	43.6	45.6	40.2	41.0	25.8
Yes	29.5	28.5	24.9	17.4	29.5	12.0	9.8	13.2	7.4	6.8
*P* value	0.003	0.000	0.000	14.2	0.003	0.154	0.133	0.000	0.908	0.687
Fever										
No	22.3	18.9	17.2	13.8	10.6	32.9	31.7	32.6	32.3	23.0
Yes	28.1	25.5	21.5	14.9	11.0	22.7	23.8	20.8	16.1	9.6
*P* value	0.002	0.000	0.000	0.411	0.718	0.974	0.544	0.153	0.913	0.156
Cough										
No	24.0	21.3	17.7	14.5	11.1	32.4	29.6	32.6	35.0	24.3
Yes	25.7	22.2	20.7	13.5	9.6	23.2	25.8	20.9	13.3	8.3
*P* value	0.325	0.337	0.003	0.497	0.227	0.343	0.026	0.473	0.679	0.756

Size at birth showed that children who were born with an average size declined from 63.6% in 1992 to 49.6% in 2015–16; with corresponding increases in the percentage of children whose size at birth was large and decreases in the percentage of children whose size at birth was small. Across all the surveys, about one in five children had diarrhoea two weeks prior to the surveys whereas almost 4 in 10 children (each) had a fever and cough two weeks prior to the survey.

**Figure 3.**
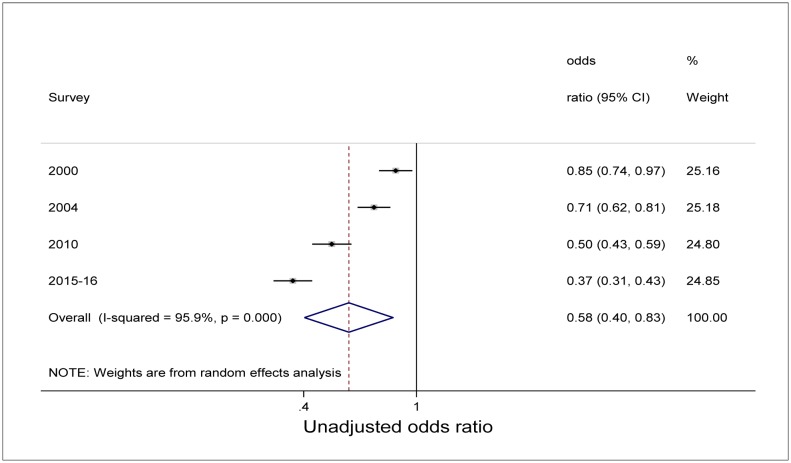
Forest plot of unadjusted relative odds of underweight among children under-5 years from Malawi demographic and health surveys conducted from 1992 to 2015–16.

**Figure 4.**
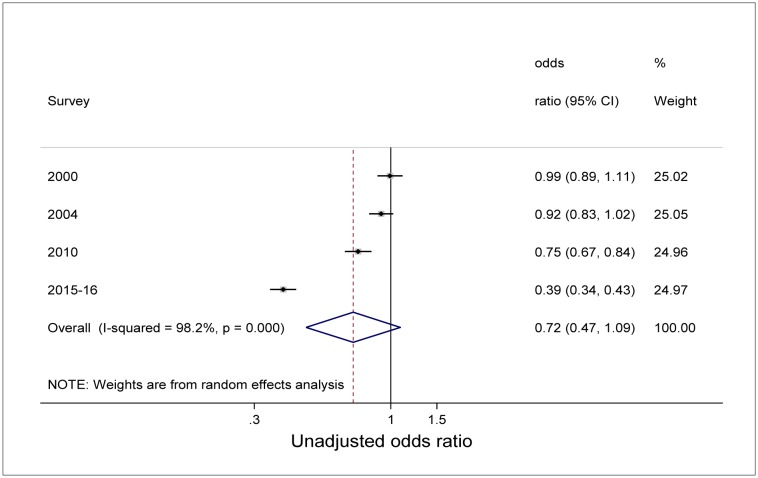
Forest plot of unadjusted relative odds of stunting among children under-5 years from Malawi demographic and health surveys conducted 1992 to 2015–16.

Unadjusted bivariate analysis of pooled data using the survey as a variable and 1992 as a reference category showed a significant decline in the odds of being underweight with time (Figure 3). Children were less likely to be underweight in 2000, 2004, 2010, and 2015–16 compared with 1992 (*P*<0.05). Similar unadjusted analysis for stunting showed also a decline in the odds of being stunted. However, while the decline was statistically significant for 2010 and 2015–16 survey period (*P*<0.000), the decline was not statistically significant in 2000 and 2004 compared with 1992 as reference category (Figure 4). Overall (unadjusted odds), underweight was 42% less likely to occur with time among all children than during early years [Odds ratio (OR) 0.58, 95%CI: 0.40-0.83] (Figure 3). Though not statistically significant, results showed that for stunting, children were 28% less likely to be stunted with time compared to early years of the survey (OR 0.72, 95%CI: 0.47-1.09) (Figure 4).

Table 4 presents logistic regression results of underweight and stunting adjusted for selected variables. For underweight, results show that children residing in central and southern regions were more likely to be underweight than children residing in northern region (OR: 1.22; 95%CI: 1.07-1.40 and OR 1.32; 95%CI: 1.15-1.50), respectively. Children from households in the middle wealth group were 20% less likely to be underweight (OR: 0.80; 95%CI: 0.71-0.90) than children from households belonging to the poorest wealth group. Similarly, children from richer households were 28% less likely to be underweight (OR: 0.72; 95%CI: 0.63-0.81) than children from the poorest wealth group; whereas those from the richest households were 54% less likely to be underweight (OR: 0.46; 95%CI: 0.38-0.55) than children from the poorest wealth group. Children of mothers with at least secondary schooling were 28% less likely to be underweight than children of mothers with no formal schooling (OR: 0.72; 95%CI: 0.59-0.87). Children whose mothers were aged 20–30 years were 16% less likely to be underweight than children whose mothers were aged under 20 years (OR: 0.84; 95%CI: 0.71-0.99). The results also showed underweight differences by sex: female children were 20% less likely to be underweight than male children (OR: 0.80; 95%CI: 0.74-0.86). Children from each of the age group (from 7–12 months to the oldest age category, 36–59 months) were more likely to be underweight than children from the youngest age group. The ORs ranged from 1.73 (7–12 months) to 1.98 (24–35 months). Children whose size at birth was average were 46% less likely to be underweight (OR: 0.54; 95%CI: 0.49-0.60) than children with small size at birth; and children who were large at birth were 62% less likely to be underweight than children who had small size at birth (OR: 0.38; 95%CI: 0.34-0.43). Having diarrhoea (OR: 1.30; 95%CI: 1.19-1.42) and fever (OR: 1.18; 95%CI: 1.09-1.27) during the two weeks preceding the survey was associated with increased odds of underweight compared with children who had no diarrhoea and fever. Children included in the latest survey rounds were less likely to be underweight than those interviewed during the earliest survey round in 1992. Thus, by 2015–16, children were 60% less likely to be underweight than children in the 1992 survey (OR: 0.40; 95%CI: 0.32-0.83). These odds were a reduction from the odds of underweight observed in 2004 (OR=0.78) and in 2010 (OR=0.61).

**Table 4.** Logistic regression of underweight and stunting on selected determinants, Malawi Demographic and Health Survey, 1992 to 2015–16.

	**Underweight (weight-for-age < -2 SD)**		**Stunting (height-for-age < -2 SD)**	
**Variable**	**OR**	**95%CI**	**OR**	**95%CI**
**Household characteristics**				
Residence				
Urban (ref)	1.00	--	1.00	--
Rural	1.04	[0.87, 1.23]	1.12	[0.99, 1.26]
Region				
Northern (ref)	1.00	--	1.00	--
Central	1.22	[1.07, 1.40]	1.26	[1.14, 1.39]
Southern	1.32	[1.15, 1.50]	1.09	[0.99, 1.20]
Wealth index				
Poorest (ref)	1.00	--	1.00	--
Poor	0.90	[0.80, 1.01]	0.87	[0.78, 0.96]
Middle	0.80	[0.71, 0.90]	0.83	[0.75, 0.92]
Richer	0.72	[0.63, 0.81]	0.70	[0.63, 0.78]
Richest	0.46	[0.38, 0.55]	0.48	[0.42, 0.54]
Source of drinking water				
Non-improved (ref)	1.00	--	1.00	--
Improved	1.02	[0.92, 1.13]	1.03	[0.95, 1.12]
Toilet facilities				
No toilet facility (ref)	1.00	--	1.00	--
Non-improved	0.90	[0.80, 1.01]	0.96	[0.87, 1.06]
Improved	0.85	[0.70, 1.04]	0.94	[0.80, 1.10]
**Maternal characteristics**				
Highest education level				
No education (ref)	1.00	--	1.00	--
Primary	0.92	[0.85, 1.00]	0.93	[0.86, 1.01]
Secondary+	0.72	[0.59, 0.87]	0.68	[0.59, 0.74]
Mothers age (yr)				
<20 (ref)	1.00	--	1.00	--
20–30	0.84	[0.71, 0.99]	0.97	[0.85, 1.12]
>30	0.87	[0.71, 1.07]	0.91	[0.76, 1.08]
Number of under-5 children living with mother				
1 (ref)	1.00	--	1.00	--
2–3	0.93	[0.80, 1.08]	0.97	[0.86, 1.09]
4–5	1.02	[0.83, 1.26]	0.98	[0.83, 1.16]
≥6	0.99	[0.80, 1.26]	0.96	[0.79, 1.17]
**Child-related characteristics**				
Sex				
Male (ref)	1.00	--	1.00	--
Female	0.80	[0.74, 0.86]	0.78	[0.73, 0.83]
Age (mo)				
<=6 (ref)	1.00	--	1.00	--
7–12	1.73	[1.47, 2.03]	1.72	[1.51, 1.96]
13–23	1.93	[1.67, 2.22]	4.59	[4.08, 5.17]
24–35	1.98	[1.71, 2.28]	5.91	[5.22, 6.68]
36–59	1.74	[1.51, 2.00]	5.02	[4.47, 5.63]
Birth order				
1 (ref)	1.00	--	1.00	--
2–3	0.98	[0.85, 1.13]	0.91	[0.82, 1.02]
≥4	0.99	[0.82, 1.20]	0.94	[0.81, 1.11]
Size at birth				
Small (ref)	1.00	--	1.00	--
Average	0.54	[0.49, 0.60]	0.70	[0.64, 0.76]
Large	0.38	[0.34, 0.43]	0.55	[0.50, 0.61]
Diarrhoea				
No (ref)	1.00	--	1.00	--
Yes	1.30	[1.19, 1.42]	1.09	[1.00, 1.18
Fever				
No (ref)	1.00	--	1.00	--
Yes	1.18	[1.09, 1.27]	1.00	[0.94, 1.07]
Cough				
No (ref)	1.00	--	1.00	--
Yes	0.99	[0.91, 1.07	1.01	[0.95, 1.07]
Survey round				
1992 (ref)	1.00	--	1.00	--
2000	0.91	[0.79, 1.05]	0.99	[0.88, 1.12]
2004	0.78	[0.68, 0.89]	0.94	[0.83, 1.05]
2010	0.61	[0.52, 0.73]	0.75	[0.66, 0.86]
2015–16	0.40	[0.32, 0.83]	0.36	[0.30, 0.43]
**N**	**29,906**		**28,419**	
**Prob > F**	**0.0000**		**0.0000**	

Note: Ref: Reference category.

Similar logistic regression results of stunting show that children residing in central region were more likely to be stunted than children residing in northern region (OR: 1.26; 95%CI: 1.14-1.39). Children from households in poor wealth group were 13% less likely to be stunted than children in the poorest wealth group (OR: 0.87; 95%CI: 0.78-0.96) whereas children in the middle wealth group were 17% less likely to be stunted (OR: 0.83; 95%CI: 0.75-0.92) than children from households belonging to the poorest wealth group. Similarly, children from richer households were 30% less likely to be stunted (OR: 0.70; 95%CI: 0.63-0.78) than children from the poorest wealth group; whereas children from the richest households were 52% less likely to be stunted (OR: 0.48; 95%CI: 0.42-0.54) than children from the poorest wealth group. Children of mothers with at least secondary schooling were 32% less likely to be stunted than children of mothers with no formal schooling (OR: 0.68; 95%CI: 0.59-0.74). Female children were 22% less likely to be stunted than male children (OR: 0.78; 95%CI: 0.73-0.83). Children from each of the age group (from 7–12 months to the oldest age category, 36–59 months) were more likely to be stunted than children from the youngest age group. The ORs ranged from 1.72 (7–12 months) to 5.91 (24–35 months). Children whose size at birth was average were 30% less likely to be stunted (OR: 0.70; 95%CI: 0.64-0.76) than children with small size at birth; and children who were large at birth were 45% less likely to be stunted than children who had small size at birth (OR: 0.55; 95%CI: 0.50-0.61). Children included in the latest survey rounds were less likely to be stunted than those interviewed during the earliest survey round in 1992. Thus, by 2015–16, children were 64% less likely to be stunted than children in the 1992 survey (OR: 0.36; 95%CI: 0.30-0.43). These odds were a reduction from the odds of stunting observed in 2010 (OR=0.75).

## 4. Discussion

The current study reports a slow decline in under nutrition rates among children under-5 in Malawi. The decline of underweight prevalence by 14 percentage points between 1992 and 2015–2016; and 23 percentage points for stunting during the same period represents an average annual reduction rate of 0.6% and 1.0%, respectively. This trend indicates the slow progress in improving child nutritional status in Malawi. The data reported in this study confirm that Malawi fell short of the national set targets in improving nutritional outcomes particularly for stunting. For example, the 2007–2011 Malawi National Nutrition Policy and Strategic Plan [11] had a target of 40% for stunting in 2011 which was lower than the observed prevalence of 48% based on the results from the 2010 DHS. However, the country achieved the target set for underweight at 15% compared with the observed prevalence of 14.1% from the 2010 DHS. The slow decline in chronic under-nutrition among under-5 children has also been reported in other sub-Saharan African countries such as Kenyav *et al*. [12] and Nigeria *et al.* [13].

The present study also documented significant levels of low size at birth of babies across all surveys; indicating a greater likelihood of mothers' poor nutritional status. Alderman and Behrman [14] highlighted the relationship between low birth weight and cognitive as well as neurological impairment among low birth weight babies; including the risk of stunting. The observed low size at birth in this study was also significantly associated with underweight and stunting. Poor cognitive and neurological development among children is associated with lower productivity and poor educational and economic outcomes, among others. Similar findings on the association between maternal under-nutrition and fetal growth restriction were also reported by Black *et al.* [15] in a global assessment of maternal and child under-nutrition.

The current findings support the literature on the negative correlation between under nutrition among children and wealth [12]. Also notable in the study are significant levels of underweight and stunting by region of residence where underweight was more pronounced in the central and the southern regions than in the northern region. Stunting was also more pronounced in the central region than the northern region. Some of the reasons for the health and socioeconomic advantage of the northern region compared with the central and the southern regions have been documented elsewhere (*e.g.*, [16]). In brief, the relative advantage of the northern region reflect intergenerational effects of education compared with the southern and central regions of Malawi whose cumulated education effects may be lower than those of the north.

The link between socio economic factors and under-nutrition has been observed in this study with the poorest households registering almost twice as much stunting and underweight compared with the richest household in all the five surveys. However, all surveys indicate reduction of both stunting and low birth weight by half. This finding emphasizes the importance of interventions which target reduction of food insecurity at the household level. Food insecurity is associated with unavailability of food, insufficient purchasing power, inappropriate distribution, or inadequate use of food which are factors that are related to poverty levels. Apart from lack of nutritious food, child malnutrition is also caused by other factors such as by frequent illness, poor care practices and lack of access to health; these factors can well be tackled within the domain of improved economic status.

Also noticeable in this analysis is the association between stunting and underweight and maternal education. All the surveys register significant proportions of stunting and underweight with the least educated mothers registering the worst outcomes, emphasizing the need for interventions to improve maternal education. In our study, we observed that underweight and stunting was less likely to occur among girls than boys; a finding that may be linked to biological differences and the role of environmental factors in influencing health and mortality outcomes [17]. The prevalence of both underweight and stunting rises significantly after >6 months of age. This could be an indication of inappropriate complementary feeding practices, cumulative effects of illness and lack of access to health care which seem to last even at 60 mo. A systematic review utilizing data from 147 countries concluded that poor breastfeeding and complementary feeding practices are some factors associated with stunting and underweight among children under-5 [18]. Maleta *et al.* [8] also argued that inappropriate complementary feeding was one significant contributor to stunting among children in rural Malawi.

Consistent with earlier studies, we found significant association between stunting, and diarrhoea and non-improved water sources across all the surveys. Although the prevalence of both stunting and underweight declined during the last two survey rounds (2010 and 2015–16), the proportions are still significantly high. The high prevalence of diarrhoea could be due to the use of un-chlorinated or contaminated water. More improved sanitary and water sources as well as better health seeking behaviour would result in improved nutritional outcomes, especially in reductions in stunting. Similar findings were also reported in Uganda [19] and Tanzania [20]. Again, improved water sources and sanitary facilities could be associated with reduced transmission of diarrhoea, hence the lower prevalence of underweight among children who had no diarrhoea [20].

Preventing stunting requires interventions that address, among other factors, the complex array of factors such as the quality and quantity of foods, reductions in the incidence and prevalence of diseases, educational outcomes, and maternal factors linked to birthweight and preterm delivery. While the evidence base on some of the potential interventions is limited [21], there are nutrition-specific interventions whose evidence in reducing the prevalence of stunting is proven and robust particularly among pregnant women and mothers of infants and young children. These include: ➀ antenatal micronutrient supplementation, which are low-cost and easily administered to improve birth outcomes and the risk of stunting [22]; ➁ Promoting consumption of healthy, diversified diets, including high-quality, nutrient-rich food in the complementary feeding period (6–24 mo); ➂ strengthened and concerted efforts targeted towards improved nutrition for pregnant mothers to reduce intra-uterine growth retardation; and ➃ intermittent presumptive treatment of malaria in malaria endemic countries like Malawi [23].

For infants and young children, proven interventions include 100,000 international units (IU) of vitamin A for infants 6–11 months of age and 200,000 IU of vitamin A every four to six months for children 12–59 months in settings with a night-blindness prevalence of 1% or higher among children aged 24–59 months or where vitamin A deficiency is at least 20% infants and children aged 6–59 months [23]. Prophylactic zinc supplementation is an ideal intervention associated with increased mean height and decreased diarrheal incidence in children [24]. Other key interventions include public provision of complementary food for children which has the potential to reduce stunting by 67% in food-insecure settings [25].

Interventions to improve nutritional and health outcomes among children under-5 can only be effective if the Government and stakeholders make more investments towards addressing gender gaps through women empowerment and efforts such as increased education access to ensure adequate knowledge and ability to access food, provide better child feeding practices and care. Government and stakeholders should also enhance efforts to implement evidence-based policies on food nutritional supplementary interventions. Partnership between the government and the research community is inevitable in order to identify new strategies and technologies to address complex nutritional problems such as stunting and anaemia.

## 5. Conclusion

This study confirms that stunting and underweight remain significant public health problems in Malawi. We note that the high levels of stunting call for measures directed towards long term investments in interventions that lead to improved energy intake, weight gain, improved nutritional outcomes, and overall health outcomes. Community-based interventions that promote access to better water, sanitation and hygiene facilities remain vital and are the pillars of hope to achieve SGD 2, target 2.2 (ending all forms of malnutrition focused on stunting, wasting and overweight among children <5 yr of age) by 2030.
